# Oxytocin Receptor Gene Polymorphism in Lactating Dogs

**DOI:** 10.3390/ani11113099

**Published:** 2021-10-29

**Authors:** Asahi Ogi, Valentina Naef, Filippo Maria Santorelli, Chiara Mariti, Angelo Gazzano

**Affiliations:** 1 Department of Veterinary Sciences, University of Pisa, 56124 Pisa, Italy; chiara.mariti@unipi.it (C.M.); angelo.gazzano@unipi.it (A.G.); 2 Neurobiology and Molecular Medicine, IRCCS Stella Maris, 56128 Calambrone, Italy; valentina.naef@gmail.com (V.N.); filippo3364@gmail.com (F.M.S.)

**Keywords:** behavior, dog, gene, maternal care, oxytocin, polymorphism, receptor

## Abstract

**Simple Summary:**

Oxytocin is commonly known for its role in mammalian bonding. Several studies have proved that polymorphisms of the oxytocin receptor gene are related to complex social behaviors in humans, but studies on the possible correlation between canine social behavior and oxytocin are mainly focused on the human–dog bond, and there are no data on the possible correlation between oxytocin receptor gene polymorphism and the maternal behavior of this species. Since mother–litter interactions could have a severe impact in determining later behavior in domestic dogs, the aim of this work was to investigate the possible correlation between salivary oxytocin, maternal care and the one known single-nucleotide polymorphism (*rs*8679684) located in the untranslated regulatory region of the oxytocin receptor gene in 19 lactating Labrador Retriever dogs. A significant correlation between oxytocin receptor gene polymorphism, peripheral oxytocin and maternal behavior in dogs was found. This implies that a more functional oxytocinergic system would lead to better mothering in dogs.

**Abstract:**

Genetic variations in the oxytocinergic system, known to regulate social behavior throughout the evolution of mammals, are believed to account for differences in mammalian social behavior. Particularly, polymorphic variants of the oxytocin receptor (*OXTR*) gene have been associated with behavioral variations in both humans and dogs. In this study, we offered evidence of the correlation between levels of salivary oxytocin (sOXT), maternal behavior and a single-nucleotide gene variant in *OXTR* (*rs*8679684) in nineteen lactating Labrador Retriever dogs. Carriers of at least one copy of the minor A allele showed higher levels of sOXT and maternal care in comparison with the homozygous T allele carriers. Considering the relevance of mother care in newborn development, these findings could help us to better understand the possible impact of variants in the *OXTR* gene in selecting dams.

## 1. Introduction

Oxytocin (OXT) is a nonapeptide hormone and neuromodulator, primarily produced in the hypothalamus from which it is secreted into both the bloodstream and the brain [1]. On the one hand, the OXT hormone stimulates uterine contractions during parturition and promotes milk ejection during lactation [2]. On the other hand, OXT is also a relevant neuromodulator of social behaviors [3]. Oxytocin is evolutionarily conserved [4], with OXT-like hormones being present in all vertebrates [5]. Isotocin, the bony fish homolog of mammalian OXT, maintains the same functions of OXT in modulating both reproductive and social behavior in osteichthyes such as zebrafish, a widely used animal model [6]. Thanks to this peculiarity, the oxytocinergic system has become widely investigated in normal and abnormal human social behavior, such as autism spectrum disorders [7], and there a growing body of literature focusing on oxytocin and its functions has emerged in the last decade [8].

Research on possible correlations between canine social behavior and OXT are mainly focused on the human–dog bond, and it is well established that salivary OXT (sOXT) in canines tends to increase after social contact with the owner or familiar persons [9,10,11]. On the contrary, the results on urinary and plasma OXT levels are still contradictory [12,13]. Since collecting saliva is a low-stress sampling method, many studies in canine species determine salivary OXT levels with different methodologies validated in dogs [9,14].

The oxytocin receptor (*OXTR*) gene polymorphism and methylation seem to be involved in regulating human social behavior [15,16,17]. The oxytocinergic system is closely related to affiliative behaviors. Common *OXTR* gene polymorphisms have been found to contribute to the development of social cognition impairments in humans, both with [7] and without autism spectrum disorder [18]. The *OXTR* DNA methylation, in turn, has been correlated with changes in *OXTR* expression. Specifically, increased *OXTR* DNA methylation seems to be associated with a reduced *OXTR* expression and, consequently, with social cognition impairments [17].

Three types of polymorphic gene variants in *OXTR* have been detected and investigated in association studies: tandem repeat polymorphism; copy-number variations; and single nucleotide polymorphisms (SNPs) [19]. In humans, in addition to social behavior, SNPs analysis has led to further exploration of genetic influence on disease susceptibility and drug sensitivity [20]. In domestic dogs, Cimarelli et al. suggested the presence of a codable association between epigenetic modification of *OXTR* and social behavior [21]. Moreover, variants in *OXTR* may have played a role in their evolution from wolves [22], and SNPs in the *OXTR* gene seem to be associated with human-directed social behavior [23] and dog–owner attachment [24]. On the contrary, Ottenheimer-Carrier et al. [25] did not find any relationship between dog genotypes and owner-reported personality assessment.

The amount of maternal care seems to have a severe impact on the emotional development of pups [26,27,28]. This phenomenon could be partially explained by the fact that DNA methylation and the expression of OXT and glucocorticoid receptor genes of the infants have been found to be susceptible to the behavior of dams toward their offspring [29,30,31].

Despite the peripheral role of OXT, it is crucial in both parturition and lactation, and despite the fact that the central role of this neuromodulator is well studied in the maternal behavior of many mammals [32,33], we are aware of a single study analyzing the influence of peripheral OXT in canine maternal behavior [34]. Therefore, the present study aimed to fill the lack of literature on this topic by investigating the possible correlation between *OXTR* gene polymorphism, peripheral OXT and maternal behavior in dogs. Specifically, we investigated two SNPs in the 3′ untranslated region (3′-UTR) of the *OXTR* gene: the known *rs*8679684 (T/A) and the novel 19,131 (A/G), recently found by Kis et al. [23].

## 2. Materials and Methods

### 2.1. Subjects

Nineteen lactating Labrador Retriever dogs (mean ± standard deviation age = 53 ± 23 months) raised by the same breeder were included. All dams nursed their puppies in a quiet whelping room without the presence of other dogs or any uncontrolled social contact. The whelping boxes employed in this study were very similar in size and layout. All of them provided the mother with the possibility of leaving the box without the puppies being able to follow her.

### 2.2. Sample Collection

Saliva samples were collected from the mothers every 3 days, from day 3 of lactation until day 21, and salivary OXT (sOXT) concentrations were measured using a Cayman Chemical ELISA Kit^®^ (Item #500440) (Ann Arbor, MI, USA), an assay previously validated in dogs [14]. Assay range = 5.9–750 pg/mL. Sensitivity = 20 pg/mL (80% Bound/Maximum Bound). Intra-assay coefficient of variation (CV%) < 9.6% (range: 46.9–370 pg/mL) and inter-assay CV% < 12.4% (range: 46.9–375 pg/mL). Each sample was analyzed in duplicate. Collection of saliva samples was performed as described in [34].

### 2.3. Behavior Analysis

After each saliva sample, the litters were videotaped for 15 min as soon as the dams were back from their walk. Each video was analyzed through a continuous sampling method using BORIS^®^ v. 7.8 [35], following a specific ethogram of maternal behavior (Table 1) previously reported in [34].

### 2.4. SNPs Genotyping

Genomic DNA from saliva was isolated by incubating 500 µL of each sample in 500 µL lysis solution containing 0.2 g/L Proteinase K, 0.1 M NaCl, 0.5% SDS and 0.01 M Tris buffer, pH = 8 at 57 °C overnight, followed by RNase treatment at room temperature. Proteins were removed with saturated NaCl (2:1 volume ratio). After the standard procedure of DNA precipitation with isopropanol and ethanol, the pellet was resuspended in 30 µL of 5 mM Tris pH = 8, 0.5 mM EDTA. The amount and purity of the DNA was determined by spectrophotometry. The range of the DNA concentration was 10–100 ng/µL. Polymerase chain reaction (PCR) was used to amplify the *rs*8679684 and 19131 (A/G) SNPs. The sequence of the dog *OXTR* gene was obtained from Ensembl (http://www.ensembl.org/, accessed on 12 April 2021) database, accession number ENSCAFG00000005553. The reaction mixture for the PCR reactions was performed with 30 ng DNA in a volume of 25 μL in a reaction mixture containing 1 μM each primer (forward primer 5′-CTCCTGGACCTATCATTTCACTCC-3′; reverse primer 5′-TTGGCTGCCTATGCCAAATG-3′) and 12.5 μL of DreamTaq PCR Master mix (2×) (Thermo Fisher Scientific, Waltham, MA, USA). The samples were heated initially to 95 °C for 2 min, each cycle comprising denaturation at 95 °C for 30 s. Primer annealing was performed at the specific temperature for 30 s and polymerization at 72 °C for 2 min. The samples were subjected to 35 cycles of amplification followed by final extension of 72 °C for 7 min. Gel electrophoresis with 1.0% agarose gel was used to confirm the success of PCR. The obtained PCR products were cleaned by ExoSAP-IT™ PCR Product Cleanup Reagent and sequenced in both forward and reverse directions with the same PCR primers. SNPs were identified by aligning and comparing the sequence data online using the ClustalW program (available at http://www.genome.jp/tools/clustalw/, accessed on 12 April 2021).

### 2.5. Statistical Analysis

The statistical analysis was performed using GraphPad Prism 7 software (GraphPad Software, Inc., San Diego, CA, USA). Normality of the data was assessed using the Shapiro–Wilk normality test. The significance between groups was determined using Tukey’s multiple comparisons test or the non-parametric one-tailed Mann–Whitney rank sum test. The values are expressed as mean ± standard error of the mean (SEM). Statistical significance was set at * *p* ≤ 0.05, ** *p* ≤ 0.01.

## 3. Results

We investigated frequencies and the Hardy–Weinberg equation of the genotype. The prevalence of the *rs*8679684 genotypes was AA (n = 2, 10.5%), AT (n = 5, 26.3%) and TT (n = 12, 63.2%). The frequency of the T allele and A allele was, respectively, 0.76 and 0.24 (χ^2^ = 1.4060).

**Figure 1:**
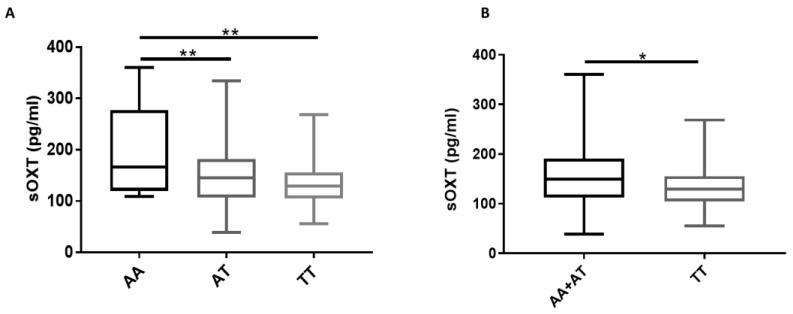
The correlation between salivary OXT (sOXT) and *OXTR* gene *rs*8679684 polymorphism: (**A**) carriers of the AA genotype (n = 2) showed higher levels of sOXT compared to carriers of the AT (n = 5) and TT (n = 12) genotype (** *p* ≤ 0.01—Tukey’s multiple comparisons test). (**B**) The presence of the A allele in SNP *rs*8679684 was associated with higher level of sOXT (* *p* ≤ 0.05—Mann–Whitney test).

Due to the limited number of dogs involved in this study, no AA genotype subjects were found for the 19,131 (A/G) SNP (AG: n = 6, 32%; GG: n = 13, 68%; AA: n = 0, 0%), so we focused our analysis only on the *rs*8679684 SNP. In comparison to the carriers of the common TT genotype, carriers of the rare AA genotype showed a higher concentration of sOXT on average (*p* = 0.002) (Figure 1A). Considering the low prevalence of the A allele, rare homozygote genotypes (AA) were grouped together with heterozygotes (AT) for the comparison with the TT genotype, as in [23]. We observed that dogs homozygous for the T allele (TT) showed lower levels of sOXT than dogs with one or two copies of the A allele (AA and AT) (*p* = 0.0345) (Figure 1B).

**Figure 2:**
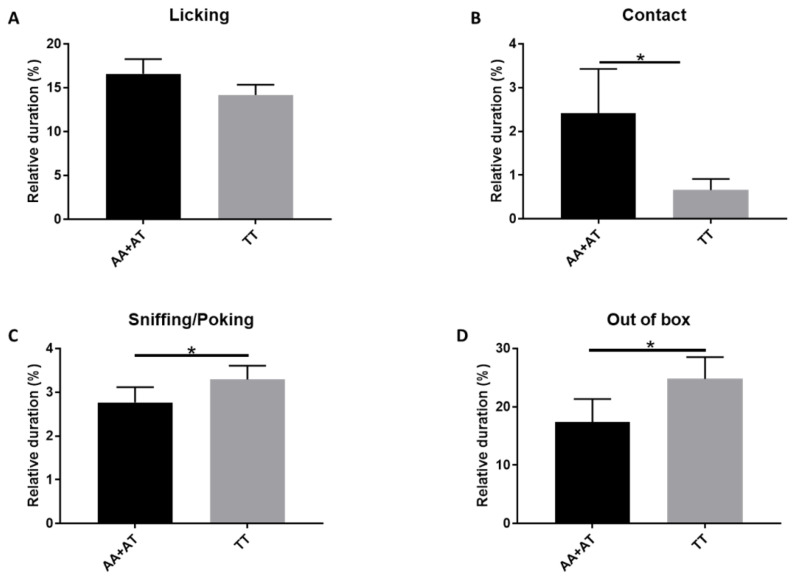
The correlation between *OXTR* gene polymorphism and relative duration (%) of maternal behaviors: (**A**) Licking, (**B**) Contact, (**C**) Sniffing/poking, (**D**) Out of box. The presence of the A allele (AA + AT) in *rs*8679684 SNP was associated with a higher level of licking (*p* = 0.1870) and contact (*p* = 0.0468), but with a lower level of sniffing/poking (*p* = 0.0374) and out of box (*p* = 0.0432) (* *p* ≤ 0.05—Mann–Whitney test).

Moreover, testing the correlation between *rs*8679684 genotype and maternal behaviors (Table 1), we found that the presence of the A allele (AA + AT) was associated, although not significantly, with a higher level of licking behavior (Figure 2A) and with a higher amount of contact (*p* = 0.0468) (Figure 2B). On the contrary, the presence of the A allele was associated with a lower level of sniffing/poking (*p* = 0.0377) (Figure 2C) and a lower amount of time spent out of box (*p* = 0.0432) (Figure 2D). Retrieving behavior was excluded from our analyses because it was expressed by only two dogs who harbored different genotypes.

**Figure 3:**
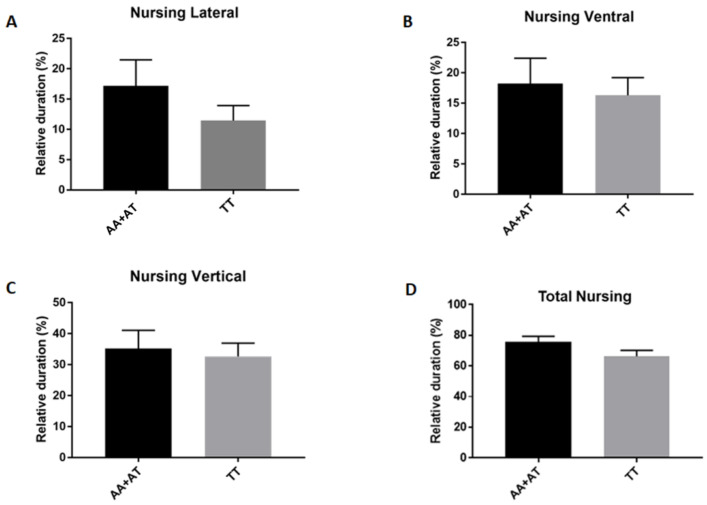
The A allele dams (AA + AT) tended to nurse more than homozygous T allele dams: (**A**) Nursing in lateral position, (**B**) Nursing in ventral position, (**C**) Nursing in vertical position, (**D**) Total Nursing = Nursing Lateral + Ventral + Vertical. The presence of the A allele (AA + AT) in *rs*8679684 SNP was associated with a higher level of nursing lateral *p* = 0.2873, nursing ventral *p* = 0.4470, nursing vertical *p* = 0.2681 and total nursing *p* = 0.49319 (*p*-values were calculated using Mann–Whitney test).

Finally, we did not observe a significant correlation between *rs*8679684 allele or genotype frequency and nursing (total, lateral, ventral and vertical), though the A allele dams tended to nurse more than those with the T allele ones (Figure 3).

## 4. Discussion

There are several SNPs annotated in the *OXTR* gene [39], and none were found to be related to the phenotypic effect such as modified gene expression. Variants that affect noncoding regulatory regions of the genome, such as 3′-UTR introns, can have modest and subtle biological effects [40]. Previous studies in humans demonstrated that a certain allele of polymorphisms located in the 3′-UTR could be associated with the increase in mRNA stability and with the increase in the amount of the receptor protein by altering miRNA binding [41,42]. In turn, an increased amount of a receptor protein expressed could indirectly influence the expression of up/downstream genes involved in the synthesis of hormones [43].

Under this hypothesis, an increased amount of *OXTR* could result in increased expression of peripheral oxytocin. The *rs*8679684 (T/A) and the 19,131 (A/G) SNPs are located in the 3′-UTR regulatory region of the *OXTR* gene [23,39], which could affect the amount of protein present. The absence of the AA genotype for the 19,131 (A/G) SNP did not allow us to include this genetic marker in the present study. Therefore, we focused on the *rs*8679684 (T/A) SNP. We did not perform a gene expression profile to determine the effect of this SNP at a cellular level in dogs with different genotypes. However, we observed that the rare AA genotype characterized the dams with a higher amount of sOXT compared to AT and TT, but these data should be interpreted cautiously considering the low prevalence of the AA genotypes (10.5%). By grouping A carriers (mono or bi-allelic) and comparing them with TT carriers, we observed a trend toward association with the peripheral levels of OXT, data that have already been reported by others [44]. In particular, the presence of the A allele in *rs*8679684 SNP was found to be related to higher amounts of sOXT in dogs.

Our findings indicate that the presence of the minor A allele in *rs*8679684 SNP could be associated with higher quality of maternal care in canine species. We found a trend in A allele-mothers (carriers of the AA + AT genotypes) to lick and nurse the puppies more than the T allele-mothers. The presence of the A allele was also significantly associated with a higher amount of time spent in contact with the offspring. On the contrary, A allele-mothers showed a lower level of sniffing/poking and a lower amount of time spent out of the box. These data suggest that the A allele-mothers provide higher quantity of maternal care except for sniffing/poking behavior. Consistent with a previous study showing a negative correlation between sniffing/poking behavior and sOXT [34], our results corroborate the hypothesis that excessive sniffing/poking behavior could be a sign of distress associated with lactation.

Despite the limited sample size of the present study, the genotype of the *rs*8679684 SNP was consistent with previous research in domestic dogs [39]. Moreover, the frequency of the minor A allele (*p* = 0.24) reflected the frequency of the two breeds—Border Collie and German Shepherd—previously investigated [23,45,46].

Despite the fact that the nineteen lactating dogs belonged to the same breed, which ensured a highly uniform sample, and despite the fact that genetic heterogeneity within dog breeds is limited [47], it is difficult to make reliable inferences on the entire canine species. Kis et al., in fact, reported an opposite effect of the polymorphism on a dog’s friendliness depending on the breed [23]. German Shepherd carriers of the A allele in *rs*8679684 achieved higher scores on the Friendliness scale compared to T allele carriers, a result contradicting opposite findings in Border Collie carriers [23]. Further studies investigating at the possible breed effect on the genetic basis of maternal behavior are needed to clarify this topic. Specifically, a genome-wide association study (GWAS) would allow us to better understand the allelic variation that underlies both peripheral OXT and maternal behavior in dogs [48,49]. Indeed, the candidate gene approach is a widely diffused method to assess non-pathological conditions in canine species, but it may have some limitations in comparison with the GWAS approach [50].

Our data have a broader implication in social sciences, maternal tutoring and even for early steps of human neurodevelopmental behavior. Previous research on dopamine D4 receptor [47] and tyrosine hydroxylase gene polymorphism [51], in addition to the abovementioned study [23], showed the significance of domestic dogs as a model species in behavioral genetics. The millenary coevolution with humans [52] and the unique history of domestication [53] gave domestic dogs the potential to mimic many human-like social abilities [54], and gave us the opportunity to link the present study with similar findings in humans. In fact, *OXTR* gene polymorphism in humans was found to predict plasma OXT, and homozygous parents for the T allele in the *OXTR* gene *rs*1042778 SNP were found to provide less parental touch than parents carrying the G allele [44]. Moreover, the *OXTR* gene *rs*53576 and *rs*1042778 in humans were found to be associated with positive parenting [55].

The present study in combination with the “biobehavioral feedback loop” [44]—a loop proposing that mother–infant contact increases the expression of OXT receptors [30], while the intracerebroventricular administration of OXT induces maternal behavior [56]—and in combination with the “allostatic theory” [4]—a theory implying that the oxytocinergic system adjusts physiological setpoints promoting adaptation—lend support to the hypothesis that a more functional oxytocinergic system could improve individual fitness, because it would lead to better parenting, which in turn would lead to a more functional oxytocinergic system in offspring. This allostatic loop could have a significant impact on selecting dams, given that maternal care is crucial in determining later behavior in domestic dogs [34].

## 5. Conclusions

Our study provides the first evidence that the presence of the A allele in *rs*8679684 SNP correlated with higher levels of sOXT and appears to be relevant in positively affecting the maternal behavior of lactating Labrador Retriever dogs. With the limitations of this study, we believe that the uniformity of participating dogs strengthened the significance of the present findings, opening a new view in the genetic background of maternal behavior and in selecting dams. Further GWAS could corroborate the assumptions made in the present research, filling the gaps between the possible conflicting findings on this topic.

## Figures and Tables

**Table 1:** Ethogram. The catalogue of maternal behaviors observed.

Ethogram		
Behavior	Definition	References
Out of box	The mother had her legs out of the whelping box not providing maternal care	[34]
Contact	The mother was lying in the whelping box with elbows on the ground and in physical contact (tail and limbs excluded) with at least one pup	[36]
Licking	The mother was licking at least one pup	Modified from [36]
Sniffing/poking	The mother was sniffing, poking, or moving at least one pup around with the nose	Modified from [36]
Retrieving	The mother was carefully carrying in her jaws at least one pup	Modified from [37]
Nursing Lateral	The mother was nursing (at least one pup suckling) while lying on her side or back, so that part, or all, of her nipples were exposed	Modified from [38]
Nursing Ventral	The mother was nursing (at least one pup suckling) while lying on her stomach, so that her nipples were not easily exposed to the puppies	Modified from [38]
Nursing Vertical	The mother was nursing (at least one pup suckling) while standing or sitting in the whelping box	Modified from [38]
Total Nursing	Nursing lateral + nursing ventral + nursing vertical. Nursing positions are mutually exclusive	Modified from [36]
Other	Any activity not assessable or not included in the behavioral catalogue	

## Data Availability

The data presented in this study are available on request from the corresponding author.
